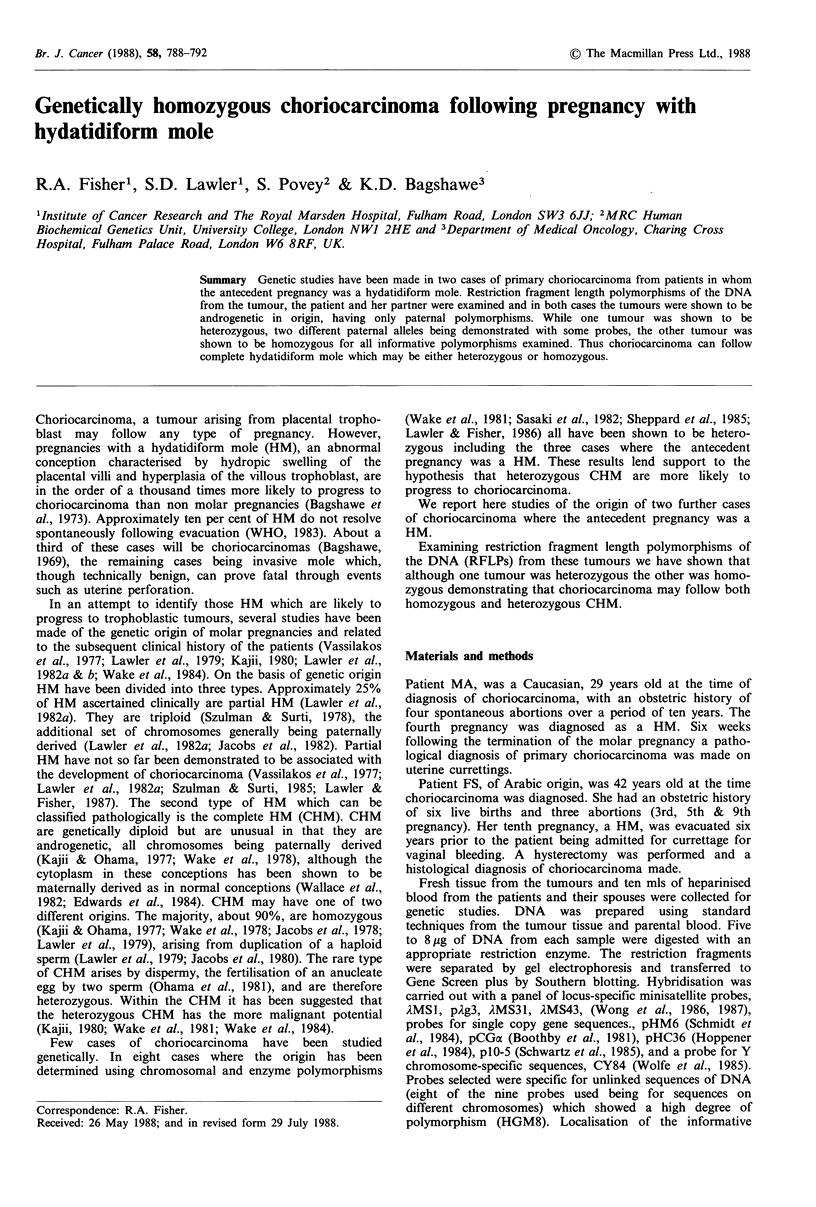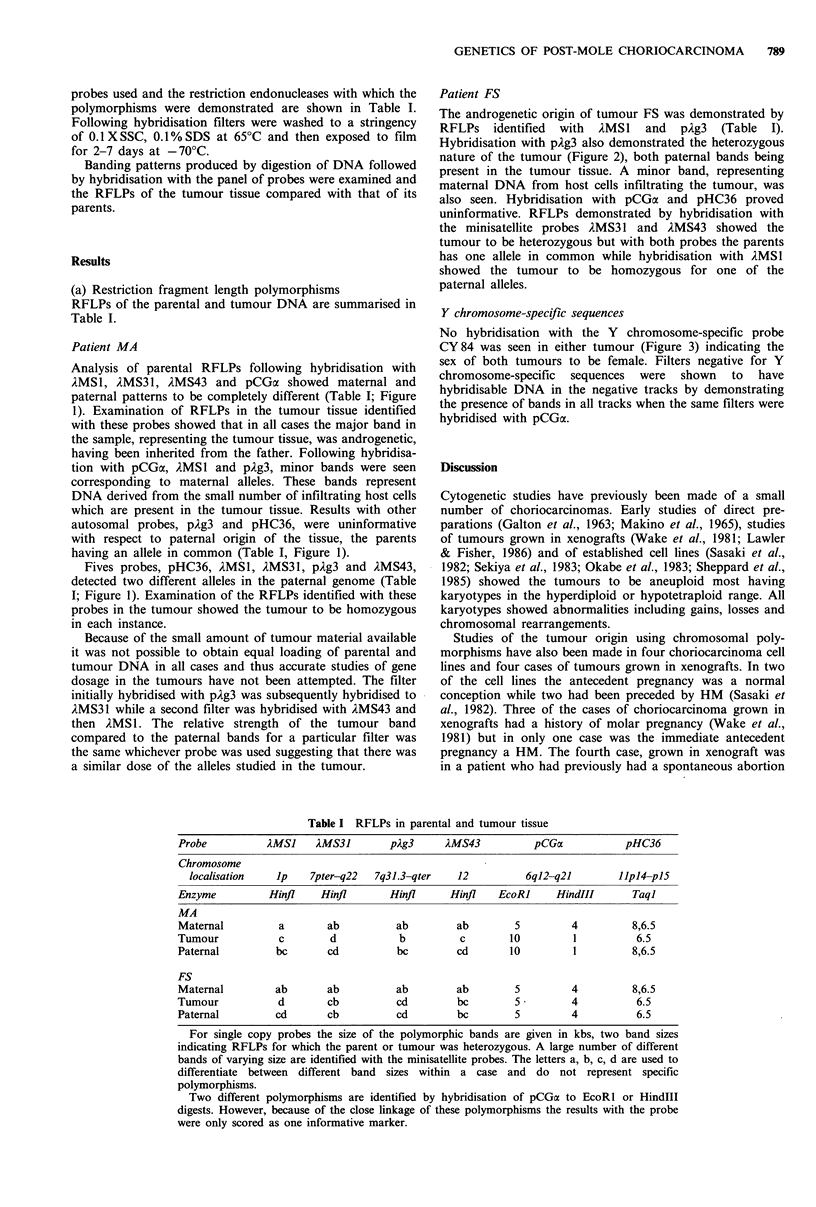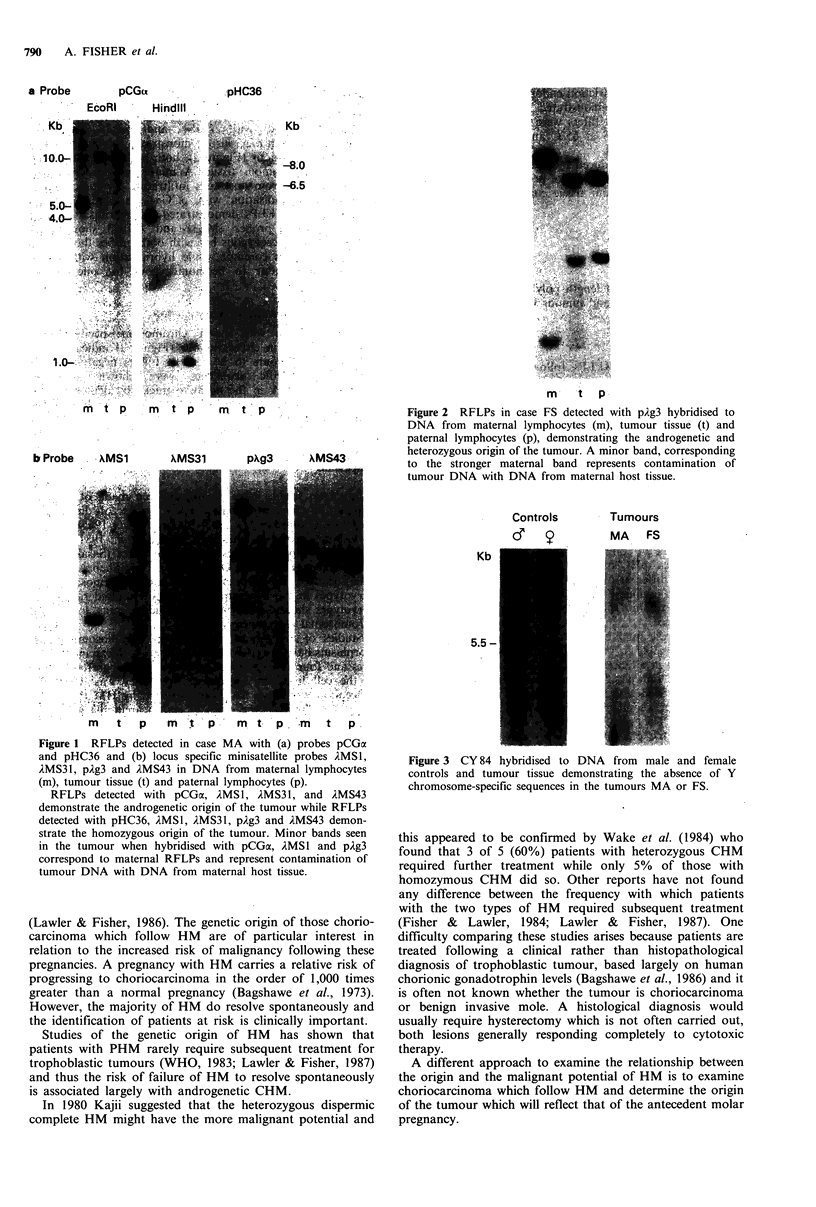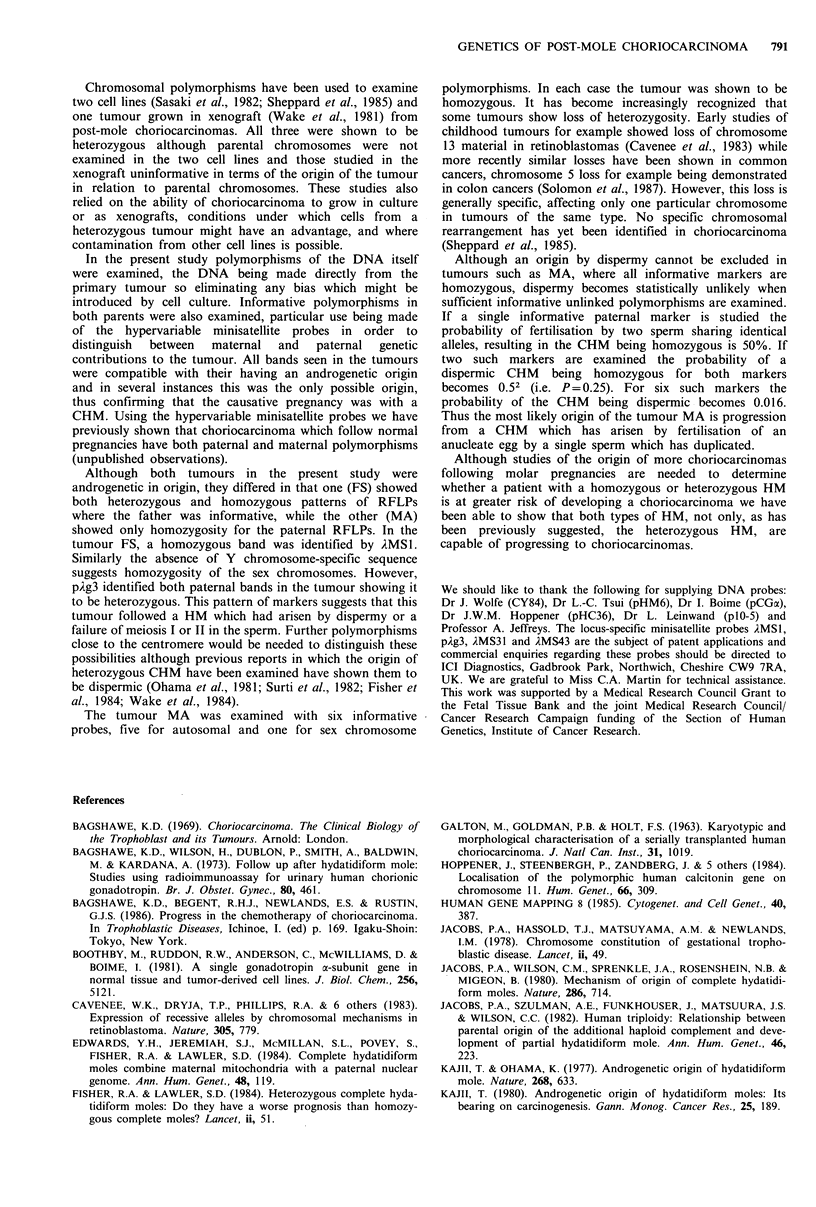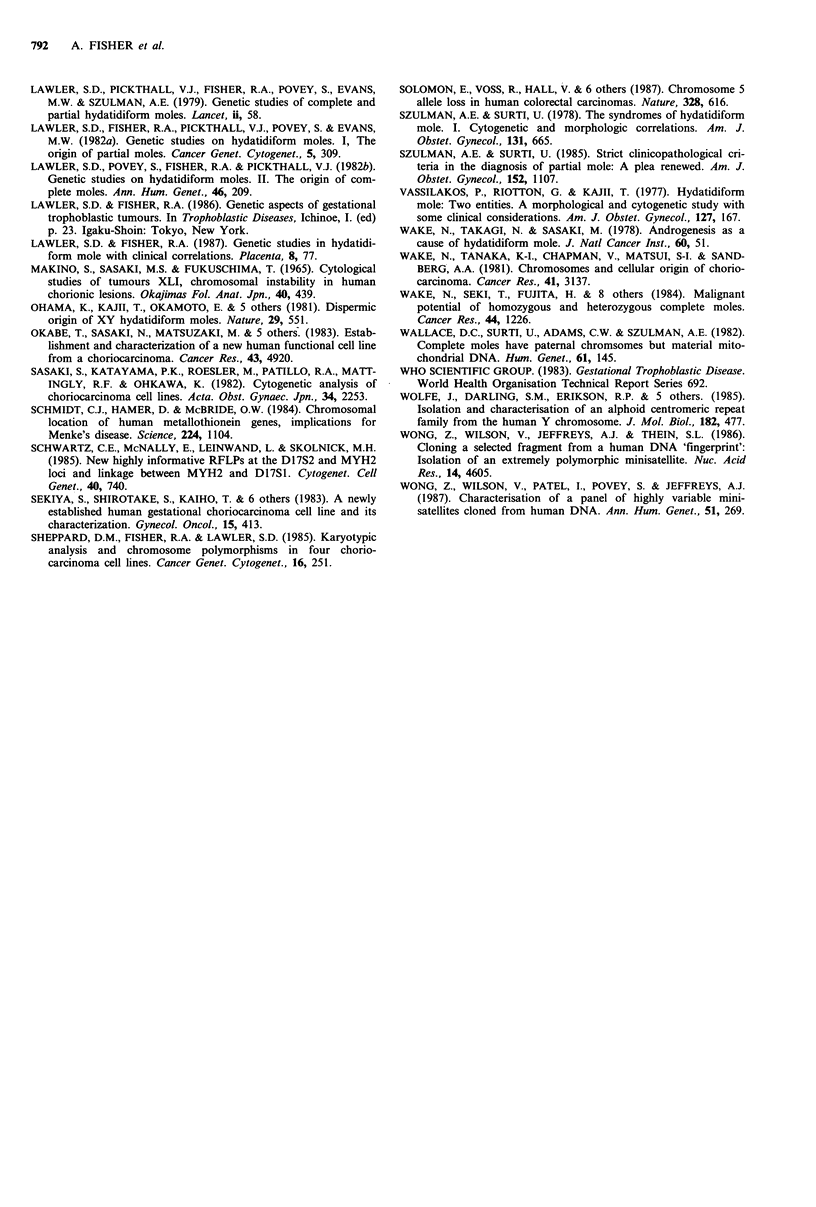# Genetically homozygous choriocarcinoma following pregnancy with hydatidiform mole.

**DOI:** 10.1038/bjc.1988.310

**Published:** 1988-12

**Authors:** R. A. Fisher, S. D. Lawler, S. Povey, K. D. Bagshawe

**Affiliations:** Institute of Cancer Research, Royal Marsden Hospital, London, UK.

## Abstract

**Images:**


Br. J. Cancer (1988), 58, 788-792 © The Macmillan Press Ltd., 1988

### Genetically homozygous choriocarcinoma following pregnancy with hydatidiform mole

R.A. Fisher[1], S.D. Lawler[1], S. Povey[2] & K.D. Bagshawe[3]

[1]*Institute of Cancer Research and The Royal Marsden Hospital, Fulham Road, London SW3 6JJ;* [2]*MRC Human Biochemical Genetics Unit, University College, London NW1 2HE and* [3]*Department of Medical Oncology, Charing Cross Hospital, Fulham Palace Road, London W6 8RF, UK.*

**Summary** Genetic studies have been made in two cases of primary choriocarcinoma from patients in whom the antecedent pregnancy was a hydatidiform mole. Restriction fragment length polymorphisms of the DNA from the tumour, the patient and her partner were examined and in both cases the tumours were shown to be androgenetic in origin, having only paternal polymorphisms. While one tumour was shown to be heterozygous, two different paternal alleles being demonstrated with some probes, the other tumour was shown to be homozygous for all informative polymorphisms examined. Thus choriocarcinoma can follow complete hydatidiform mole which may be either heterozygous or homozygous.

Choriocarcinoma, a tumour arising from placental trophoblast may follow any type of pregnancy. However, pregnancies with a hydatidiform mole (HM), an abnormal conception characterised by hydropic swelling of the placental villi and hyperplasia of the villous trophoblast, are in the order of a thousand times more likely to progress to choriocarcinoma than non molar pregnancies (Bagshawe *et al.*, 1973). Approximately ten per cent of HM do not resolve spontaneously following evacuation (WHO, 1983). About a third of these cases will be choriocarcinomas (Bagshawe, 1969), the remaining cases being invasive mole which, though technically benign, can prove fatal through events such as uterine perforation.

In an attempt to identify those HM which are likely to progress to trophoblastic tumours, several studies have been made of the genetic origin of molar pregnancies and related to the subsequent clinical history of the patients (Vassilakos *et al.*, 1977; Lawler *et al.*, 1979; Kajii, 1980; Lawler *et al.*, 1982*a* & *b*; Wake *et al.*, 1984). On the basis of genetic origin HM have been divided into three types. Approximately 25% of HM ascertained clinically are partial HM (Lawler *et al.*, 1982*a*). They are triploid (Szulman & Surti, 1978), the additional set of chromosomes generally being paternally derived (Lawler *et al.*, 1982*a*; Jacobs *et al.*, 1982). Partial HM have not so far been demonstrated to be associated with the development of choriocarcinoma (Vassilakos *et al.*, 1977; Lawler *et al.*, 1982*a*; Szulman & Surti, 1985; Lawler & Fisher, 1987). The second type of HM which can be classified pathologically is the complete HM (CHM). CHM are genetically diploid but are unusual in that they are androgenetic, all chromosomes being paternally derived (Kajii & Ohama, 1977; Wake *et al.*, 1978), although the cytoplasm in these conceptions has been shown to be maternally derived as in normal conceptions (Wallace *et al.*, 1982; Edwards *et al.*, 1984). CHM may have one of two different origins. The majority, about 90%, are homozygous (Kajii & Ohama, 1977; Wake *et al.*, 1978; Jacobs *et al.*, 1978; Lawler *et al.*, 1979), arising from duplication of a haploid sperm (Lawler *et al.*, 1979; Jacobs *et al.*, 1980). The rare type of CHM arises by dispermy, the fertilisation of an anucleate egg by two sperm (Ohama *et al.*, 1981), and are therefore heterozygous. Within the CHM it has been suggested that the heterozygous CHM has the more malignant potential (Kajii, 1980; Wake *et al.*, 1981; Wake *et al.*, 1984).

Few cases of choriocarcinoma have been studied genetically. In eight cases where the origin has been determined using chromosomal and enzyme polymorphisms (Wake *et al.*, 1981; Sasaki *et al.*, 1982; Sheppard *et al.*, 1985; Lawler & Fisher, 1986) all have been shown to be heterozygous including the three cases where the antecedent pregnancy was a HM. These results lend support to the hypothesis that heterozygous CHM are more likely to progress to choriocarcinoma.

We report here studies of the origin of two further cases of choriocarcinoma where the antecedent pregnancy was a HM.

Examining restriction fragment length polymorphisms of the DNA (RFLPs) from these tumours we have shown that although one tumour was heterozygous the other was homozygous demonstrating that choriocarcinoma may follow both homozygous and heterozygous CHM.

### Materials and methods

Patient MA, was a Caucasian, 29 years old at the time of diagnosis of choriocarcinoma, with an obstetric history of four spontaneous abortions over a period of ten years. The fourth pregnancy was diagnosed as a HM. Six weeks following the termination of the molar pregnancy a pathological diagnosis of primary choriocarcinoma was made on uterine currettings.

Patient FS, of Arabic origin, was 42 years old at the time choriocarcinoma was diagnosed. She had an obstetric history of six live births and three abortions (3rd, 5th & 9th pregnancy). Her tenth pregnancy, a HM, was evacuated six years prior to the patient being admitted for currettage for vaginal bleeding. A hysterectomy was performed and a histological diagnosis of choriocarcinoma made.

Fresh tissue from the tumours and ten mls of heparinised blood from the patients and their spouses were collected for genetic studies. DNA was prepared using standard techniques from the tumour tissue and parental blood. Five to 8 $\mu g$ of DNA from each sample were digested with an appropriate restriction enzyme. The restriction fragments were separated by gel electrophoresis and transferred to Gene Screen plus by Southern blotting. Hybridisation was carried out with a panel of locus-specific minisatellite probes, $\lambda$MS1, p$\lambda$g3, $\lambda$MS31, $\lambda$MS43, (Wong *et al.*, 1986, 1987), probes for single copy gene sequences., pHM6 (Schmidt *et al.*, 1984), pCG$\alpha$ (Boothby *et al.*, 1981), pHC36 (Hoppener *et al.*, 1984), p10-5 (Schwartz *et al.*, 1985), and a probe for Y chromosome-specific sequences, CY84 (Wolfe *et al.*, 1985). Probes selected were specific for unlinked sequences of DNA (eight of the nine probes used being for sequences on different chromosomes) which showed a high degree of polymorphism (HGM8). Localisation of the informative

Correspondence: R.A. Fisher.
Received: 26 May 1988; and in revised form 29 July 1988.

probes used and the restriction endonucleases with which the polymorphisms were demonstrated are shown in Table I. Following hybridisation filters were washed to a stringency of 0.1 X SSC, 0.1% SDS at 65°C and then exposed to film for 2–7 days at −70°C.

Banding patterns produced by digestion of DNA followed by hybridisation with the panel of probes were examined and the RFLPs of the tumour tissue compared with that of its parents.

### Results

(a) Restriction fragment length polymorphisms
RFLPs of the parental and tumour DNA are summarised in Table I.

*Patient MA*

Analysis of parental RFLPs following hybridisation with λMS1, λMS31, λMS43 and pCGα showed maternal and paternal patterns to be completely different (Table I; Figure 1). Examination of RFLPs in the tumour tissue identified with these probes showed that in all cases the major band in the sample, representing the tumour tissue, was androgenetic, having been inherited from the father. Following hybridisation with pCGα, λMS1 and pλg3, minor bands were seen corresponding to maternal alleles. These bands represent DNA derived from the small number of infiltrating host cells which are present in the tumour tissue. Results with other autosomal probes, pλg3 and pHC36, were uninformative with respect to paternal origin of the tissue, the parents having an allele in common (Table I, Figure 1).

Fives probes, pHC36, λMS1, λMS31, pλg3 and λMS43, detected two different alleles in the paternal genome (Table I; Figure 1). Examination of the RFLPs identified with these probes in the tumour showed the tumour to be homozygous in each instance.

Because of the small amount of tumour material available it was not possible to obtain equal loading of parental and tumour DNA in all cases and thus accurate studies of gene dosage in the tumours have not been attempted. The filter initially hybridised with pλg3 was subsequently hybridised to λMS31 while a second filter was hybridised with λMS43 and then λMS1. The relative strength of the tumour band compared to the paternal bands for a particular filter was the same whichever probe was used suggesting that there was a similar dose of the alleles studied in the tumour.

*Patient FS*

The androgenetic origin of tumour FS was demonstrated by RFLPs identified with λMS1 and pλg3 (Table I). Hybridisation with pλg3 also demonstrated the heterozygous nature of the tumour (Figure 2), both paternal bands being present in the tumour tissue. A minor band, representing maternal DNA from host cells infiltrating the tumour, was also seen. Hybridisation with pCGα and pHC36 proved uninformative. RFLPs demonstrated by hybridisation with the minisatellite probes λMS31 and λMS43 showed the tumour to be heterozygous but with both probes the parents has one allele in common while hybridisation with λMS1 showed the tumour to be homozygous for one of the paternal alleles.

*Y chromosome-specific sequences*

No hybridisation with the Y chromosome-specific probe CY 84 was seen in either tumour (Figure 3) indicating the sex of both tumours to be female. Filters negative for Y chromosome-specific sequences were shown to have hybridisable DNA in the negative tracks by demonstrating the presence of bands in all tracks when the same filters were hybridised with pCGα.

### Discussion

Cytogenetic studies have previously been made of a small number of choriocarcinomas. Early studies of direct preparations (Galton *et al.*, 1963; Makino *et al.*, 1965), studies of tumours grown in xenografts (Wake *et al.*, 1981; Lawler & Fisher, 1986) and of established cell lines (Sasaki *et al.*, 1982; Sekiya *et al.*, 1983; Okabe *et al.*, 1983; Sheppard *et al.*, 1985) showed the tumours to be aneuploid most having karyotypes in the hyperdiploid or hypotetraploid range. All karyotypes showed abnormalities including gains, losses and chromosomal rearrangements.

Studies of the tumour origin using chromosomal polymorphisms have also been made in four choriocarcinoma cell lines and four cases of tumours grown in xenografts. In two of the cell lines the antecedent pregnancy was a normal conception while two had been preceded by HM (Sasaki *et al.*, 1982). Three of the cases of choriocarcinoma grown in xenografts had a history of molar pregnancy (Wake *et al.*, 1981) but in only one case was the immediate antecedent pregnancy a HM. The fourth case, grown in xenograft was in a patient who had previously had a spontaneous abortion

**Table I** RFLPs in parental and tumour tissue

| *Probe* | *λMS1* | *λMS31* | *pλg3* | *λMS43* | *pCGα* | | *pHC36* |
|---|---|---|---|---|---|---|---|
| *Chromosome localisation* | *1p* | *7pter–q22* | *7q31.3–qter* | *12* | *6q12–q21* | | *11p14–p15* |
| *Enzyme* | *HinfI* | *HinfI* | *HinfI* | *HinfI* | *EcoR1* | *HindIII* | *TaqI* |
| *MA* | | | | | | | |
| Maternal | a | ab | ab | ab | 5 | 4 | 8,6.5 |
| Tumour | c | d | b | c | 10 | 1 | 6.5 |
| Paternal | bc | cd | bc | cd | 10 | 1 | 8,6.5 |
| *FS* | | | | | | | |
| Maternal | ab | ab | ab | ab | 5 | 4 | 8,6.5 |
| Tumour | d | cb | cd | bc | 5 | 4 | 6.5 |
| Paternal | cd | cb | cd | bc | 5 | 4 | 6.5 |

For single copy probes the size of the polymorphic bands are given in kbs, two band sizes indicating RFLPs for which the parent or tumour was heterozygous. A large number of different bands of varying size are identified with the minisatellite probes. The letters a, b, c, d are used to differentiate between different band sizes within a case and do not represent specific polymorphisms.

Two different polymorphisms are identified by hybridisation of pCGα to EcoR1 or HindIII digests. However, because of the close linkage of these polymorphisms the results with the probe were only scored as one informative marker.

**Figure 1** RFLPs detected in case MA with (a) probes pCGα and pHC36 and (b) locus specific minisatellite probes λMS1, λMS31, pλg3 and λMS43 in DNA from maternal lymphocytes (m), tumour tissue (t) and paternal lymphocytes (p).

RFLPs detected with pCGα, λMS1, λMS31, and λMS43 demonstrate the androgenetic origin of the tumour while RFLPs detected with pHC36, λMS1, λMS31, pλg3 and λMS43 demonstrate the homozygous origin of the tumour. Minor bands seen in the tumour when hybridised with pCGα, λMS1 and pλg3 correspond to maternal RFLPs and represent contamination of tumour DNA with DNA from maternal host tissue.

**Figure 2** RFLPs in case FS detected with pλg3 hybridised to DNA from maternal lymphocytes (m), tumour tissue (t) and paternal lymphocytes (p), demonstrating the androgenetic and heterozygous origin of the tumour. A minor band, corresponding to the stronger maternal band represents contamination of tumour DNA with DNA from maternal host tissue.

**Figure 3** CY84 hybridised to DNA from male and female controls and tumour tissue demonstrating the absence of Y chromosome-specific sequences in the tumours MA or FS.

(Lawler & Fisher, 1986). The genetic origin of those choriocarcinoma which follow HM are of particular interest in relation to the increased risk of malignancy following these pregnancies. A pregnancy with HM carries a relative risk of progressing to choriocarcinoma in the order of 1,000 times greater than a normal pregnancy (Bagshawe *et al.*, 1973). However, the majority of HM do resolve spontaneously and the identification of patients at risk is clinically important.

Studies of the genetic origin of HM has shown that patients with PHM rarely require subsequent treatment for trophoblastic tumours (WHO, 1983; Lawler & Fisher, 1987) and thus the risk of failure of HM to resolve spontaneously is associated largely with androgenetic CHM.

In 1980 Kajii suggested that the heterozygous dispermic complete HM might have the more malignant potential and this appeared to be confirmed by Wake *et al.* (1984) who found that 3 of 5 (60%) patients with heterozygous CHM required further treatment while only 5% of those with homozymous CHM did so. Other reports have not found any difference between the frequency with which patients with the two types of HM required subsequent treatment (Fisher & Lawler, 1984; Lawler & Fisher, 1987). One difficulty comparing these studies arises because patients are treated following a clinical rather than histopathological diagnosis of trophoblastic tumour, based largely on human chorionic gonadotrophin levels (Bagshawe *et al.*, 1986) and it is often not known whether the tumour is choriocarcinoma or benign invasive mole. A histological diagnosis would usually require hysterectomy which is not often carried out, both lesions generally responding completely to cytotoxic therapy.

A different approach to examine the relationship between the origin and the malignant potential of HM is to examine choriocarcinoma which follow HM and determine the origin of the tumour which will reflect that of the antecedent molar pregnancy.

Chromosomal polymorphisms have been used to examine two cell lines (Sasaki *et al.*, 1982; Sheppard *et al.*, 1985) and one tumour grown in xenograft (Wake *et al.*, 1981) from post-mole choriocarcinomas. All three were shown to be heterozygous although parental chromosomes were not examined in the two cell lines and those studied in the xenograft uninformative in terms of the origin of the tumour in relation to parental chromosomes. These studies also relied on the ability of choriocarcinoma to grow in culture or as xenografts, conditions under which cells from a heterozygous tumour might have an advantage, and where contamination from other cell lines is possible.

In the present study polymorphisms of the DNA itself were examined, the DNA being made directly from the primary tumour so eliminating any bias which might be introduced by cell culture. Informative polymorphisms in both parents were also examined, particular use being made of the hypervariable minisatellite probes in order to distinguish between maternal and paternal genetic contributions to the tumour. All bands seen in the tumours were compatible with their having an androgenetic origin and in several instances this was the only possible origin, thus confirming that the causative pregnancy was with a CHM. Using the hypervariable minisatellite probes we have previously shown that choriocarcinoma which follow normal pregnancies have both paternal and maternal polymorphisms (unpublished observations).

Although both tumours in the present study were androgenetic in origin, they differed in that one (FS) showed both heterozygous and homozygous patterns of RFLPs where the father was informative, while the other (MA) showed only homozygosity for the paternal RFLPs. In the tumour FS, a homozygous band was identified by λMS1. Similarly the absence of Y chromosome-specific sequence suggests homozygosity of the sex chromosomes. However, pλg3 identified both paternal bands in the tumour showing it to be heterozygous. This pattern of markers suggests that this tumour followed a HM which had arisen by dispermy or a failure of meiosis I or II in the sperm. Further polymorphisms close to the centromere would be needed to distinguish these possibilities although previous reports in which the origin of heterozygous CHM have been examined have shown them to be dispermic (Ohama *et al.*, 1981; Surti *et al.*, 1982; Fisher *et al.*, 1984; Wake *et al.*, 1984).

The tumour MA was examined with six informative probes, five for autosomal and one for sex chromosome polymorphisms. In each case the tumour was shown to be homozygous. It has become increasingly recognized that some tumours show loss of heterozygosity. Early studies of childhood tumours for example showed loss of chromosome 13 material in retinoblastomas (Cavenee *et al.*, 1983) while more recently similar losses have been shown in common cancers, chromosome 5 loss for example being demonstrated in colon cancers (Solomon *et al.*, 1987). However, this loss is generally specific, affecting only one particular chromosome in tumours of the same type. No specific chromosomal rearrangement has yet been identified in choriocarcinoma (Sheppard *et al.*, 1985).

Although an origin by dispermy cannot be excluded in tumours such as MA, where all informative markers are homozygous, dispermy becomes statistically unlikely when sufficient informative unlinked polymorphisms are examined. If a single informative paternal marker is studied the probability of fertilisation by two sperm sharing identical alleles, resulting in the CHM being homozygous is 50%. If two such markers are examined the probability of a dispermic CHM being homozygous for both markers becomes $0.5^2$ (i.e. $P=0.25$). For six such markers the probability of the CHM being dispermic becomes 0.016. Thus the most likely origin of the tumour MA is progression from a CHM which has arisen by fertilisation of an anucleate egg by a single sperm which has duplicated.

Although studies of the origin of more choriocarcinomas following molar pregnancies are needed to determine whether a patient with a homozygous or heterozygous HM is at greater risk of developing a choriocarcinoma we have been able to show that both types of HM, not only, as has been previously suggested, the heterozygous HM, are capable of progressing to choriocarcinomas.

We should like to thank the following for supplying DNA probes: Dr J. Wolfe (CY84), Dr L.-C. Tsui (pHM6), Dr I. Boime (pCGα), Dr J.W.M. Hoppener (pHC36), Dr L. Leinwand (p10-5) and Professor A. Jeffreys. The locus-specific minisatellite probes λMS1, pλg3, λMS31 and λMS43 are the subject of patent applications and commercial enquiries regarding these probes should be directed to ICI Diagnostics, Gadbrook Park, Northwich, Cheshire CW9 7RA, UK. We are grateful to Miss C.A. Martin for technical assistance. This work was supported by a Medical Research Council Grant to the Fetal Tissue Bank and the joint Medical Research Council/ Cancer Research Campaign funding of the Section of Human Genetics, Institute of Cancer Research.

### References

BAGSHAWE, K.D. (1969). *Choriocarcinoma. The Clinical Biology of the Trophoblast and its Tumours.* Arnold: London.

BAGSHAWE, K.D., WILSON, H., DUBLON, P., SMITH, A., BALDWIN, M. & KARDANA, A. (1973). Follow up after hydatidiform mole: Studies using radioimmunoassay for urinary human chorionic gonadotropin. *Br. J. Obstet. Gynec.*, **80**, 461.

BAGSHAWE, K.D., BEGENT, R.H.J., NEWLANDS, E.S. & RUSTIN, G.J.S. (1986). Progress in the chemotherapy of choriocarcinoma. In *Trophoblastic Diseases*, Ichinoe, I. (ed) p. 169. Igaku-Shoin: Tokyo, New York.

BOOTHBY, M., RUDDON, R.W., ANDERSON, C., McWILLIAMS, D. & BOIME, I. (1981). A single gonadotropin α-subunit gene in normal tissue and tumor-derived cell lines. *J. Biol. Chem.*, **256**, 5121.

CAVENEE, W.K., DRYJA, T.P., PHILLIPS, R.A. & 6 others (1983). Expression of recessive alleles by chromosomal mechanisms in retinoblastoma. *Nature*, **305**, 779.

EDWARDS, Y.H., JEREMIAH, S.J., McMILLAN, S.L., POVEY, S., FISHER, R.A. & LAWLER, S.D. (1984). Complete hydatidiform moles combine maternal mitochondria with a paternal nuclear genome. *Ann. Hum. Genet.*, **48**, 119.

FISHER, R.A. & LAWLER, S.D. (1984). Heterozygous complete hydatidiform moles: Do they have a worse prognosis than homozygous complete moles? *Lancet*, **ii**, 51.

GALTON, M., GOLDMAN, P.B. & HOLT, F.S. (1963). Karyotypic and morphological characterisation of a serially transplanted human choriocarcinoma. *J. Natl Can. Inst.*, **31**, 1019.

HOPPENER, J., STEENBERGH, P., ZANDBERG, J. & 5 others (1984). Localisation of the polymorphic human calcitonin gene on chromosome 11. *Hum. Genet.*, **66**, 309.

HUMAN GENE MAPPING 8 (1985). *Cytogenet. and Cell Genet.*, **40**, 387.

JACOBS, P.A., HASSOLD, T.J., MATSUYAMA, A.M. & NEWLANDS, I.M. (1978). Chromosome constitution of gestational trophoblastic disease. *Lancet*, **ii**, 49.

JACOBS, P.A., WILSON, C.M., SPRENKLE, J.A., ROSENSHEIN, N.B. & MIGEON, B. (1980). Mechanism of origin of complete hydatidiform moles. *Nature*, **286**, 714.

JACOBS, P.A., SZULMAN, A.E., FUNKHOUSER, J., MATSUURA, J.S. & WILSON, C.C. (1982). Human triploidy: Relationship between parental origin of the additional haploid complement and development of partial hydatidiform mole. *Ann. Hum. Genet.*, **46**, 223.

KAJII, T. & OHAMA, K. (1977). Androgenetic origin of hydatidiform mole. *Nature*, **268**, 633.

KAJII, T. (1980). Androgenetic origin of hydatidiform moles: Its bearing on carcinogenesis. *Gann. Monog. Cancer Res.*, **25**, 189.

LAWLER, S.D., PICKTHALL, V.J., FISHER, R.A., POVEY, S., EVANS, M.W. & SZULMAN, A.E. (1979). Genetic studies of complete and partial hydatidiform moles. *Lancet*, **ii**, 58.

LAWLER, S.D., FISHER, R.A., PICKTHALL, V.J., POVEY, S. & EVANS, M.W. (1982*a*). Genetic studies on hydatidiform moles. I, The origin of partial moles. *Cancer Genet. Cytogenet.*, **5**, 309.

LAWLER, S.D., POVEY, S., FISHER, R.A. & PICKTHALL, V.J. (1982*b*). Genetic studies on hydatidiform moles. II. The origin of complete moles. *Ann. Hum. Genet.*, **46**, 209.

LAWLER, S.D. & FISHER, R.A. (1986). Genetic aspects of gestational trophoblastic tumours. In *Trophoblastic Diseases*, Ichinoe, I. (ed) p. 23. Igaku-Shoin: Tokyo, New York.

LAWLER, S.D. & FISHER, R.A. (1987). Genetic studies in hydatidiform mole with clinical correlations. *Placenta*, **8**, 77.

MAKINO, S., SASAKI, M.S. & FUKUSCHIMA, T. (1965). Cytological studies of tumours XLI, chromosomal instability in human chorionic lesions. *Okajimas Fol. Anat. Jpn.*, **40**, 439.

OHAMA, K., KAJII, T., OKAMOTO, E. & 5 others (1981). Dispermic origin of XY hydatidiform moles. *Nature*, **29**, 551.

OKABE, T., SASAKI, N., MATSUZAKI, M. & 5 others. (1983). Establishment and characterization of a new human functional cell line from a choriocarcinoma. *Cancer Res.*, **43**, 4920.

SASAKI, S., KATAYAMA, P.K., ROESLER, M., PATILLO, R.A., MATTINGLY, R.F. & OHKAWA, K. (1982). Cytogenetic analysis of choriocarcinoma cell lines. *Acta. Obst. Gynaec. Jpn.*, **34**, 2253.

SCHMIDT, C.J., HAMER, D. & McBRIDE, O.W. (1984). Chromosomal location of human metallothionein genes, implications for Menke's disease. *Science*, **224**, 1104.

SCHWARTZ, C.E., McNALLY, E., LEINWAND, L. & SKOLNICK, M.H. (1985). New highly informative RFLPs at the D17S2 and MYH2 loci and linkage between MYH2 and D17S1. *Cytogenet. Cell Genet.*, **40**, 740.

SEKIYA, S., SHIROTAKE, S., KAIHO, T. & 6 others (1983). A newly established human gestational choriocarcinoma cell line and its characterization. *Gynecol. Oncol.*, **15**, 413.

SHEPPARD, D.M., FISHER, R.A. & LAWLER, S.D. (1985). Karyotypic analysis and chromosome polymorphisms in four choriocarcinoma cell lines. *Cancer Genet. Cytogenet.*, **16**, 251.

SOLOMON, E., VOSS, R., HALL, V. & 6 others (1987). Chromosome 5 allele loss in human colorectal carcinomas. *Nature*, **328**, 616.

SZULMAN, A.E. & SURTI, U. (1978). The syndromes of hydatidiform mole. I. Cytogenetic and morphologic correlations. *Am. J. Obstet. Gynecol.*, **131**, 665.

SZULMAN, A.E. & SURTI, U. (1985). Strict clinicopathological criteria in the diagnosis of partial mole: A plea renewed. *Am. J. Obstet. Gynecol.*, **152**, 1107.

VASSILAKOS, P., RIOTTON, G. & KAJII, T. (1977). Hydatidiform mole: Two entities. A morphological and cytogenetic study with some clinical considerations. *Am. J. Obstet. Gynecol.*, **127**, 167.

WAKE, N., TAKAGI, N. & SASAKI, M. (1978). Androgenesis as a cause of hydatidiform mole. *J. Natl Cancer Inst.*, **60**, 51.

WAKE, N., TANAKA, K-I., CHAPMAN, V., MATSUI, S-I. & SANDBERG, A.A. (1981). Chromosomes and cellular origin of choriocarcinoma. *Cancer Res.*, **41**, 3137.

WAKE, N., SEKI, T., FUJITA, H. & 8 others (1984). Malignant potential of homozygous and heterozygous complete moles. *Cancer Res.*, **44**, 1226.

WALLACE, D.C., SURTI, U., ADAMS, C.W. & SZULMAN, A.E. (1982). Complete moles have paternal chromsomes but material mitochondrial DNA. *Hum. Genet.*, **61**, 145.

WHO SCIENTIFIC GROUP. (1983). *Gestational Trophoblastic Disease*. World Health Organisation Technical Report Series 692.

WOLFE, J., DARLING, S.M., ERIKSON, R.P. & 5 others. (1985). Isolation and characterisation of an alphoid centromeric repeat family from the human Y chromosome. *J. Mol. Biol.*, **182**, 477.

WONG, Z., WILSON, V., JEFFREYS, A.J. & THEIN, S.L. (1986). Cloning a selected fragment from a human DNA 'fingerprint': Isolation of an extremely polymorphic minisatellite. *Nuc. Acid Res.*, **14**, 4605.

WONG, Z., WILSON, V., PATEL, I., POVEY, S. & JEFFREYS, A.J. (1987). Characterisation of a panel of highly variable minisatellites cloned from human DNA. *Ann. Hum. Genet.*, **51**, 269.